# Uterine Perforation With Extrauterine Intrauterine Device (IUD) Migration Following Blunt Abdominal Trauma: A Case Report

**DOI:** 10.7759/cureus.110477

**Published:** 2026-06-08

**Authors:** Joakyna De Santiago-Pagán, Lina S Mártir-Ramírez, Christian C Colón-Vega, Jose E Martínez-Correa, Yolymar Poventud

**Affiliations:** 1 Emergency Medicine, University of Puerto Rico, Medical Sciences Campus, San Juan, PRI; 2 Surgery, University of Puerto Rico, Medical Sciences Campus, San Juan, PRI

**Keywords:** copper intrauterine device, iud migration, management of uterine perforation by iud, migrated intrauterine device, uterine perforation by intrauterine device

## Abstract

Intrauterine devices (IUDs) are widely used long-acting reversible contraceptives with a favorable safety profile. However, uterine perforation is a rare complication that may result in extrauterine migration of the IUD and may also involve adjacent pelvic or abdominal structures, and is often an incidental finding during imaging performed for unrelated indications. We present the case of a 32-year-old female with a copper IUD placed approximately seven years prior without gynecologic follow-up who presented to the emergency department following a motor vehicle collision with abdominal pain. The patient was hemodynamically stable. Abdominal examination demonstrated diffuse tenderness to palpation with involuntary guarding. Computed tomography of the abdomen and pelvis demonstrated uterine fundal perforation with extrauterine migration of the IUD abutting and possibly penetrating the distal sigmoid colon without other imaging findings. Repeat imaging at a tertiary trauma center confirmed stable extrauterine IUD positioning without evidence of interval change. Given clinical stability and absence of overt visceral perforation, multidisciplinary evaluation favored conservative inpatient observation with close outpatient follow-up for definitive removal. The absence of prior gynecologic symptoms supports blunt trauma as the precipitating mechanism of uterine perforation. Perforated IUDs do not always cause pneumoperitoneum or hemoperitoneum. Clinicians evaluating patients with indwelling IUDs after blunt pelvic trauma should maintain a high index of suspicion for acute perforation and ensure systematic assessment of device position on cross-sectional imaging.

## Introduction

Intrauterine devices (IUDs) are among the most used long-acting reversible contraceptive methods worldwide due to their high efficacy, durability, and favorable safety profile [1,2]. Despite their overall safety, complications may occur, including expulsion, abnormal uterine bleeding, pelvic pain, infection, and, less commonly, uterine perforation [3]. Uterine perforation occurs in approximately 1 to 2 per 1,000 insertions, with higher risk reported among postpartum and breastfeeding patients [3,4]. In some cases, perforation may remain clinically silent for prolonged periods and lead to delayed extrauterine migration involving adjacent pelvic or intra-abdominal structures [5].

Uterine perforation and migration are related but distinct processes. Uterine perforation refers to disruption of the uterine wall, whereas migration describes displacement of the device beyond the uterine cavity following complete or partial perforation [3,5]. Over time, migrated devices may involve adjacent pelvic and abdominal structures, including the omentum, bladder, and gastrointestinal tract, particularly the sigmoid colon, because of its close anatomic relationship to the posterior uterine wall [5,6]. In some cases, local inflammatory reactions associated with copper-containing devices may contribute to gradual transmural erosion and progressive tissue penetration [3,7]. Extrauterine IUDs have been most commonly identified in the omentum and pouch of Douglas; involvement of the sigmoid colon, while uncommon, is facilitated by its anatomic proximity to the posterior uterine wall and may progress to full-thickness penetration [5]. Notably, up to one-third of patients with migrated IUDs may remain asymptomatic at the time of diagnosis [8].

Cross-sectional imaging obtained for blunt abdominal or pelvic trauma may reveal IUD malposition or extrauterine migration directly related to the traumatic event [5,9]. Distinguishing acute trauma-precipitated perforation from a pre-existing chronic process is diagnostically challenging and carries important implications for clinical interpretation and management [9]. This distinction is further complicated by the fact that prolonged IUD retention may create predisposing myometrial changes that lower the threshold for perforation under external mechanical loading [3,6,7].

We present the case of a 32-year-old woman with a long-standing copper IUD who presented following a motor vehicle collision (MVC) with acute-onset diffuse abdominal pain and was found to have uterine perforation with extrauterine migration and possible distal sigmoid colon involvement, with clinical and historical features supporting blunt trauma as the precipitating mechanism in the setting of a predisposed myometrium.

## Case presentation

A 32-year-old female G1P1 with no significant past medical history presented to the emergency department following an MVC in which she was T-boned on the passenger side while restrained, with airbag deployment. The vehicle did not roll over, extrication was not required, and the patient remained ambulatory at the scene. Upon arrival at the emergency room, she reported diffuse abdominal pain. She denied loss of consciousness, nausea, vomiting, fever, vaginal bleeding, rectal bleeding, or genitourinary symptoms. Her gynecologic history was notable for placement of a copper IUD approximately seven years prior without subsequent gynecologic follow-up. Vital signs were within normal limits, including blood pressure 120/72 mmHg, heart rate 80 beats per minute, respiratory rate 17 breaths per minute, temperature 37°C, and oxygen saturation of 97% on room air. The patient was alert, oriented, and in no acute distress. Abdominal examination revealed a nondistended abdomen with diffuse tenderness to palpation and involuntary guarding; rebound tenderness was not elicited. No seatbelt sign or other external signs of trauma were identified. Pelvic examination revealed normal external genitalia without abnormal discharge or bleeding. IUD strings were only partially visualized, raising concern for displacement. Focused bedside ultrasonography demonstrated no free fluid within the hepatorenal, splenorenal, pelvic, or pericardial spaces and no sonographic evidence of pneumothorax.

Initial laboratory evaluation, including complete blood count, metabolic panel, coagulation studies, and serum pregnancy testing, was unremarkable except for a mild elevation in hepatic transaminases, which was attributed to abdominal trauma without evidence of progressive injury on serial evaluation (Table 1). There was no laboratory evidence of ongoing hemorrhage or end-organ dysfunction.

**Table 1:** Laboratory studies Laboratory findings at initial presentation at the referring facility and upon subsequent evaluation at the tertiary care center. BUN: Blood Urea Nitrogen, GFR: Glomerular Filtration Rate, AST: Aspartate Aminotransferase, ALT: Alanine Aminotransferase, WBC: White Blood Cell Count, RBC: Red Blood Cell Count, MCV: Mean Corpuscular Volume, PT: Prothrombin Time, PTT: Partial Thromboplastin Time, ß-hCG: Beta Human Chorionic Gonadotropin

Laboratory Test	Normal Range	Initial Results	Post-24-hour repeat
Glucose	70–99 mg/dL	100	95
BUN	7.0–17.0 mg/dL	14	10.2
Creatinine	0.52–1.04 mg/dL	0.71	0.67
BUN/Creatinine	7.0–25.0	19.7	15.3
GFR	70–95 mL/min/1.73 m²	95	119
Sodium	137–145 mmol/L	139	138
Potassium	3.5–5.1 mmol/L	4.2	3.9
Chloride	98–107 mmol/L	104	103
CO₂	22.0–30.0 mEq/L	26	26.6
Anion Gap	10.0–20.0	13.2	8
Calcium	8.40–10.2 mg/dL	8.9	9.0
AST	14.0–36.0 U/L	54	Unavailable
ALT	4–50 U/L	71	Unavailable
Alkaline Phosphatase	38–126 U/L	81	Unavailable
Total Bilirubin	0.20–1.30 mg/dL	0.36	Unavailable
Lactate	4.5–19.8 mg/dL	Unavailable	6.6
WBC	5.20–12.40 x10⁹/L	10.0	7.02
RBC	4.20–5.40 x10⁶/µL	4.40	4.50
Hemoglobin	12.0–16.0 g/dL	13.5	13.9
Hematocrit	37.0–47.0%	39.2	39.5
MCV	80.0–99.0 fL	89.2	87.8
Platelets	130–400 x10⁹/L	323	305
Neutrophils %	40.0–74.0%	67.8	48.1
Lymphocytes%	19.0-48.0%	24.3	41.5
Monocytes %	3.4–9.0%	5.6	7.3
Eosinophils %	0.0–7.0%	1.5	2.4
Basophils %	0.0–1.5%	0.8	0.7
PT	10.2–12.2 sec	11.2	11.0
PTT	23–31 sec	31.4	28.7
ß-hCG	—	Negative	—
Laboratory Test	Normal Range	Initial Results	Post-24-hour repeat
Glucose	70–99 mg/dL	100	95
BUN	7.0–17.0 mg/dL	14	10.2
Creatinine	0.52–1.04 mg/dL	0.71	0.67
BUN/Creatinine	7.0–25.0	19.7	15.3
GFR	70–95 mL/min/1.73 m²	95	119
Sodium	137–145 mmol/L	139	138
Potassium	3.5–5.1 mmol/L	4.2	3.9
Chloride	98–107 mmol/L	104	103
CO₂	22.0–30.0 mEq/L	26	26.6
Anion Gap	10.0–20.0	13.2	8
Calcium	8.40–10.2 mg/dL	8.9	9.0
AST	14.0–36.0 U/L	54	Unavailable
ALT	4–50 U/L	71	Unavailable
Alkaline Phosphatase	38–126 U/L	81	Unavailable
Total Bilirubin	0.20–1.30 mg/dL	0.36	Unavailable
Lactate	4.5–19.8 mg/dL	Unavailable	6.6
WBC	5.20–12.40 x10⁹/L	10.0	7.02
RBC	4.20–5.40 x10⁶/µL	4.40	4.50
Hemoglobin	12.0–16.0 g/dL	13.5	13.9
Hematocrit	37.0–47.0%	39.2	39.5
MCV	80.0–99.0 fL	89.2	87.8
Platelets	130–400 x10⁹/L	323	305
Neutrophils %	40.0–74.0%	67.8	48.1
Lymphocytes%	19.0-48.0%	24.3	41.5
Monocytes %	3.4–9.0%	5.6	7.3
Eosinophils %	0.0–7.0%	1.5	2.4
Basophils %	0.0–1.5%	0.8	0.7
PT	10.2–12.2 sec	11.2	11.0
PTT	23–31 sec	31.4	28.7
ß-hCG	—	Negative	—

Computed tomography (CT) of the abdomen and pelvis with intravenous contrast demonstrated malposition of the copper IUD with extrauterine extension through the uterine fundus into the pelvis (Figure 1). The posterior arm of the device abutted and possibly penetrated the distal sigmoid colon (Figure 2). No pneumoperitoneum, hemoperitoneum, bowel obstruction, or free pelvic fluid was identified; however, the absence of these findings does not exclude acute perforation [9].

**Figure 1:**
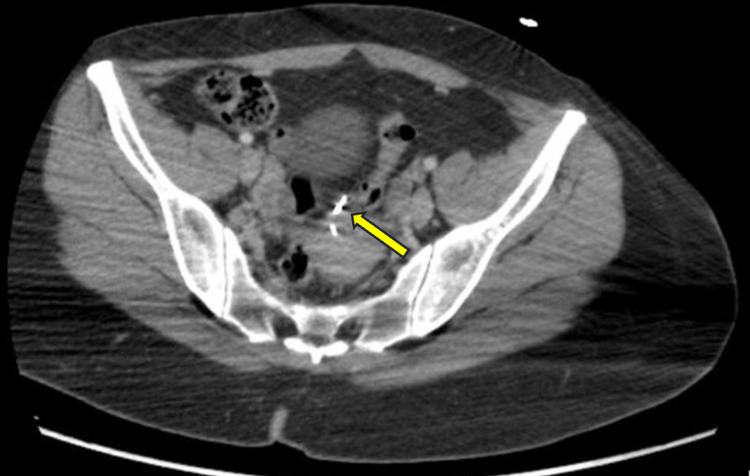
Uterine perforation with extrauterine migration of IUD Axial view of contrast-enhanced CT of the abdomen and pelvis demonstrating extrauterine extension of copper IUD (yellow arrow) extending beyond the expected endometrial cavity through the uterine fundus. A correctly positioned IUD should reside entirely within the uterine cavity; extension of the device beyond the uterine wall, as seen here, is the defining imaging feature of perforation.

**Figure 2:**
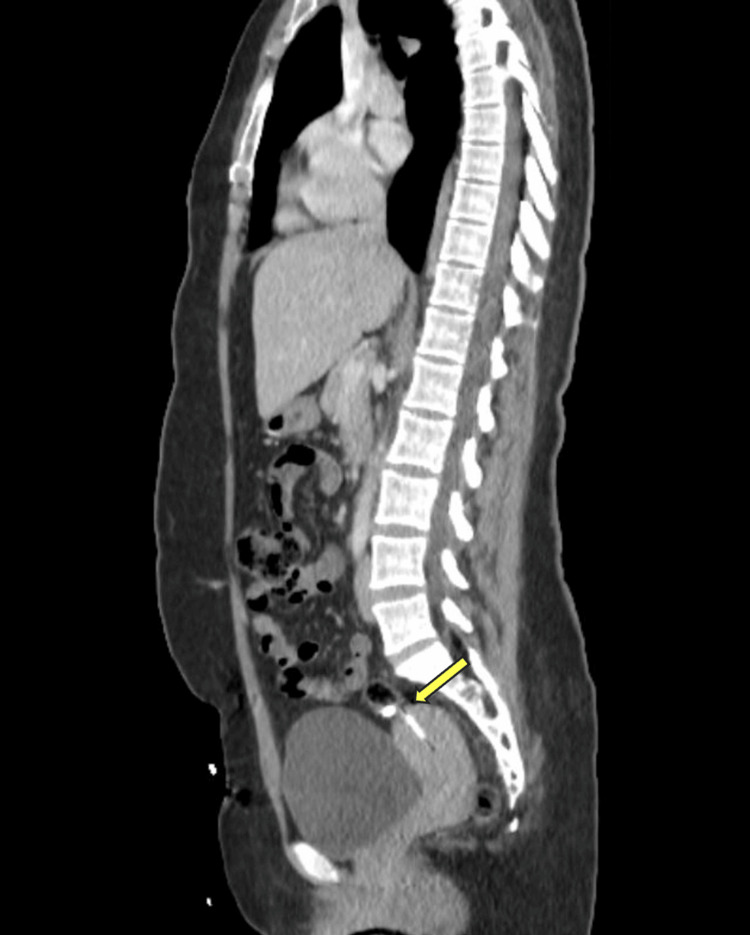
Extrauterine copper IUD with suspected transmural sigmoid colon involvement Sagittal view of contrast-enhanced CT of the abdomen and pelvis demonstrating the migrated copper IUD (yellow arrow) extending beyond the uterine serosa with close abutment of the anterior wall of the distal sigmoid colon. The proximity of the device tip to the bowel wall raises concern for transmural colonic penetration; however, the absence of pneumoperitoneum, hemoperitoneum, or pericolonic inflammatory stranding suggests a contained process without overtly disrupted bowel continuity.

Given concern for extrauterine IUD migration with possible sigmoid involvement, the patient was transferred to a tertiary trauma center for multidisciplinary evaluation and potential operative management if required.

After transfer to the tertiary care center, repeat computed tomography without intravenous contrast confirmed stable positioning of the extrauterine IUD (Figures 3, 4). The device was again visualized extending through the uterine fundus with the distal tip in close proximity to the sigmoid colon. No new traumatic intra-abdominal or pelvic injuries were identified. Imaging stability between the two studies suggested a contained rather than evolving perforation, though the precise timing of the initial uterine wall breach could not be determined from imaging alone.

**Figure 3:**
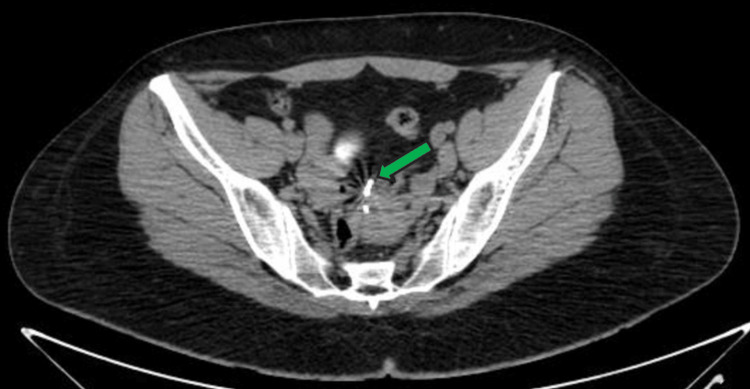
Follow-up imaging study at tertiary center Axial view of non-contrast CT of the abdomen and pelvis demonstrating the distal tip of the copper IUD extending toward the sigmoid colon (green arrow), with unchanged positioning relative to the initial study. The stability of device location between the two imaging studies argues against active or ongoing displacement from the trauma and supports a contained process. No new traumatic intra-abdominal or pelvic injury is identified.

**Figure 4:**
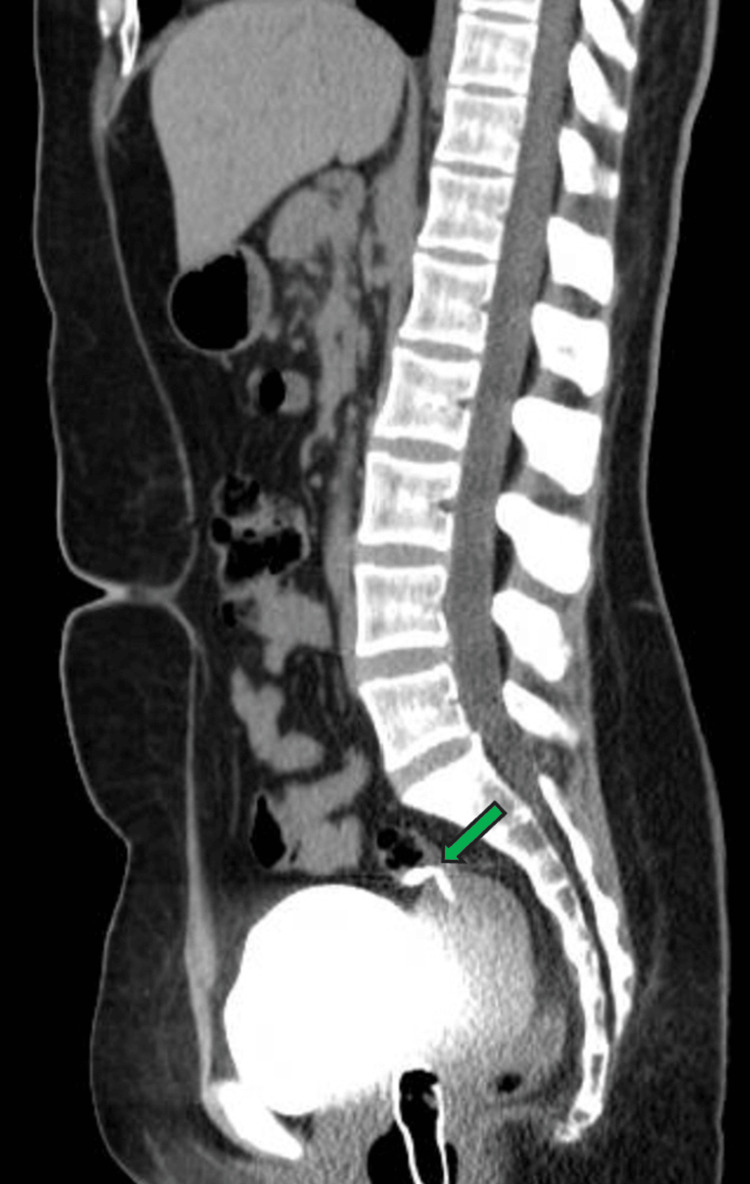
Follow-up imaging study at tertiary center Sagittal view of non-contrast CT at the tertiary trauma center confirming uterine fundal perforation with extrauterine IUD migration and suspected sigmoid apposition. The copper IUD (green arrow) is visualized traversing the uterine fundus and extending toward the anterior wall of the distal sigmoid colon. Imaging features are consistent with uterine perforation and secondary extrauterine migration; definitive characterization of transmural sigmoid involvement requires further assessment. No peritoneal free air or free fluid is identified.

The patient was admitted for observation and remained hemodynamically stable throughout hospitalization. She tolerated oral intake without difficulty and demonstrated no worsening of abdominal symptoms or evolution of peritoneal findings beyond those noted at initial evaluation. Perirectal and nasal swab cultures did not reveal multiresistant organisms. Multidisciplinary evaluation by trauma surgery and obstetrics and gynecology favored conservative inpatient management given the absence of overt bowel perforation, sepsis, obstruction, or hemodynamic instability. The patient was subsequently discharged with close outpatient gynecologic follow-up for definitive device removal and provided strict return precautions for worsening abdominal pain, fever, gastrointestinal symptoms, or abnormal vaginal bleeding.

In summary, the key diagnostic triggers in this case were: (1) a history of long-standing copper IUD without gynecologic follow-up, (2) acute-onset diffuse abdominal pain with involuntary guarding following high-energy blunt pelvic trauma, (3) partial visualization of IUD strings on pelvic examination, and (4) CT imaging demonstrating extrauterine device extension through the uterine fundus with suspected sigmoid involvement in the absence of pneumoperitoneum or hemoperitoneum. 

## Discussion

The onset of diffuse abdominal pain with involuntary guarding immediately following an MVC in a patient with a seven-year history of an indwelling copper IUD and no prior gynecologic complaints raises strong clinical suspicion for trauma-precipitated uterine perforation. Although the clinical presentation of IUD-related perforation is highly variable, with up to one-third of patients remaining asymptomatic at the time of diagnosis, the acute onset of pain in direct temporal relationship to high-energy blunt pelvic trauma argues against a preexisting, insidious process [8,10]. The complete absence of gynecologic and systemic symptoms throughout seven years of IUD use makes it unlikely that significant peritoneal or visceral involvement predated the collision. Patients with chronic or pre-existing uterine perforation and extrauterine migration, particularly with sigmoid involvement, would be expected to develop symptoms over time, including pelvic pain, dyspareunia, abnormal uterine bleeding, or changes in bowel habits [5,6]. The absence of any such complaints, combined with the acute onset of diffuse abdominal pain and involuntary guarding immediately following the collision, supports trauma as the precipitating event rather than the incidental unmasking of a longstanding process [11].

IUDs are broadly classified into two types based on their active component: copper-releasing and levonorgestrel-releasing hormonal devices. Copper IUDs exert their contraceptive effect primarily through the release of copper ions, which induce a localized sterile inflammatory reaction within the endometrium that impairs sperm motility and prevents fertilization; this mechanism also underlies their efficacy as emergency contraception [12]. Levonorgestrel-releasing IUDs act through local progestogenic effects, including thickening of cervical mucus, suppression of endometrial proliferation, and impairment of sperm transport, with long-term use resulting in endometrial atrophy [11]. On cross-sectional imaging, a correctly positioned IUD appears centrally located within the endometrial cavity, with its T-shaped frame oriented near the uterine fundus and both transverse arms extending symmetrically toward the cornua, while the stem courses inferiorly toward the internal cervical os [9]. Deviation from this configuration, including eccentric positioning, asymmetric or inferior displacement, or extension beyond the endometrial cavity, indicates malposition or embedment and warrants further evaluation [9]. It is important to note that copper IUDs are readily visible on both plain radiography and CT due to their metallic composition, whereas levonorgestrel-releasing IUDs may be less conspicuous on certain modalities [9].

The role of blunt trauma as a direct precipitant of IUD-related uterine perforation and gastrointestinal invasion, while rare, is supported by prior case reports. Davoodabadi et al. described a case in which a copper IUD, which was previously well-tolerated for two half years without symptoms, perforated the uterus and invaded the sigmoid colon following severe pelvic blunt trauma [12]. The authors concluded that trauma was either the direct cause of perforation or, at minimum, a significant contributing factor, as the absence of uterine scarring, abscess, or free pus on surgical exploration argued against a chronic underlying process [12]. Cheung et al. similarly documented that mechanical forces acting on a malpositioned or structurally compromised uterine wall can precipitate perforation and device displacement, particularly in the context of a pre-existing predisposing condition such as scarring or myometrial thinning [13]. In the present case, seven years of copper IUD indwelling may have produced localized inflammatory changes at the fundal myometrium, rendering the uterine wall susceptible to perforation under the compressive forces generated by a lateral-impact collision with airbag deployment.

Imaging findings in this case are consistent with an acute, contained perforation rather than a chronic process. Importantly, it has been established in the radiology literature that perforated IUDs do not typically produce intraperitoneal imaging findings such as pneumoperitoneum, hemoperitoneum, or peritoneal inflammatory changes, even when perforation is clinically significant [9]. The absence of these findings on both the initial and repeat CT should therefore not be interpreted as evidence against an acute mechanism. Furthermore, the lack of chronic inflammatory changes, such as omental fat stranding, adhesion formation, or bowel wall thickening around the device, that would be expected after years of extraluminal foreign body presence, is more consistent with an acute event [5,9,12]. The partial, rather than fully absent, visualization of the IUD strings on pelvic examination also suggests the possibility of recent rather than longstanding displacement of the device from its intrauterine position [5,6].

The characterization of IUD position and depth of myometrial involvement on CT carries important limitations that are directly relevant to this case. As described in the imaging literature, the most common CT finding of an embedded IUD is an eccentric position within the uterine cavity; however, CT may overcall penetration into the myometrium due to artifact generated by the copper and barium content of the device, making it difficult to determine the precise border of the IUD relative to the endometrial-myometrial interface [9]. Radiologists can be more confident of serosal involvement when the device is seen to extend near or beyond the uterine serosal surface, as was the case in the present patient [9]. In cases where partial embedment is suspected but not definitive on CT, transvaginal ultrasonography with three-dimensional reconstruction remains the modality of choice for confirmation, given its superior resolution of the endometrial-myometrial interface [14]. Notably, the degree of embedment may not be completely characterized until direct visualization at hysteroscopy is performed. Accurate description of embedment depth on preoperative imaging is essential, as it directly influences surgical planning: superficially embedded or partially perforated devices may be amenable to hysteroscopic or ultrasound-guided retrieval, whereas devices extending near or through the uterine serosa often require laparoscopic removal [9]. CT remains the primary modality for characterizing extrauterine IUD location and its anatomic relationship to adjacent structures in the emergency setting, and systematic assessment of device position should be part of any trauma CT review in patients with an indwelling device [9].

Penetration of an extrauterine IUD into adjacent hollow visceral structures, including the sigmoid colon, is facilitated by at least one of three recognized mechanisms: primary mechanical perforation at the time of insertion, gradual erosion driven by uterine contractility and bowel peristalsis, or acute displacement by an external compressive force [6,12]. In cases involving blunt abdominal or pelvic trauma, the mechanical energy transferred to the pelvis may be sufficient to drive a device already positioned at or near the uterine wall across the remaining myometrium and into adjacent structures [12]. The sigmoid colon is the most commonly involved visceral organ, given its anatomic proximity to the posterior uterine wall [5,15]. The restraint configuration of the T-bone lateral impact with contralateral-side airbag deployment would be expected to generate significant lateral compressive and torsional forces across the pelvis, plausibly capable of completing a perforation in a fundal wall that had been progressively weakened by years of copper-mediated sterile inflammation.

Prior reports have specifically emphasized that in any patient with an IUD who develops abdominal pain following pelvic blunt trauma, uterine perforation with gastrointestinal involvement must be actively evaluated with appropriate imaging [12]. Regardless of the precise mechanism, management in this case was appropriately guided by hemodynamic stability, clinical trajectory, and imaging findings rather than by the presumed chronicity of the perforation [16,17]. The absence of overt bowel perforation, sepsis, obstruction, or hemodynamic instability supported an initial non-operative approach with close multidisciplinary observation. Most extrauterine IUDs ultimately require removal due to the risk of delayed complications, including bowel injury, fistula formation, adhesions, and abscess formation [6,17]. Laparoscopic removal is generally preferred in clinically stable patients for its lower morbidity and superior visualization [17,18]. Endoscopic retrieval may be considered in confirmed cases of intraluminal gastrointestinal or urinary tract involvement [6,14].

## Conclusions

This case illustrates a presentation of uterine perforation by a copper IUD with secondary extrauterine migration and suspected transmural apposition to the sigmoid colon, identified in the context of blunt pelvic trauma. The acute onset of diffuse abdominal pain with involuntary guarding following an MVC in a patient with no prior gynecologic symptoms despite seven years of IUD use supports a trauma-precipitated rather than chronic mechanism. The absence of intraperitoneal imaging findings should not be used to exclude an acute etiology. Clinicians evaluating patients with indwelling IUDs following blunt abdominal or pelvic trauma must maintain a high index of suspicion for acute perforation and ensure systematic review of device position on cross-sectional imaging. Management should be guided by hemodynamic stability, symptom trajectory, and imaging findings. Although selected clinically stable patients without evidence of overt visceral injury may initially be managed conservatively, definitive removal of the extrauterine device remains the standard of care.
